# Mental health training program for community mental health staff in Guangzhou, China: effects on knowledge of mental illness and stigma

**DOI:** 10.1186/1752-4458-8-49

**Published:** 2014-12-04

**Authors:** Jie Li, Juan Li, Yuanguang Huang, Graham Thornicroft

**Affiliations:** Guangzhou Brain Hospital, Guangzhou Medical University, 36# Mingxin Road, Liwan, Guangzhou 510370 China; Health Service and Population Research Department, Institute of Psychiatry, King’s College London, London, SE5 8AF UK

**Keywords:** Training course, Stigma, Community mental health staff

## Abstract

**Background:**

In order to reduce the huge treatment gap in mental health, WHO has called for integrating mental health into primary care. The purposes of this study are to provide a training course to improve the community mental health staff’s knowledge of mental health and reduce stigma related to mental illness, as well as to evaluate the impact of this training on knowledge and stigma.

**Methods:**

The training intervention was a one day course for community mental health staff in Guangzhou, China. Evaluation questionnaires were given before and after the training session. Mental health knowledge was assessed using two vignettes. Stigma was evaluated by the Mental Illness: Clinicians’ Attitudes Scale (MICA) and the Reported and Intended Behavior Scale (RIBS).

**Results:**

A total of 99 community mental health staff from eight regions in Guangzhou, China were recruited for the study. The training course did not lead to a significant improvement of participants’ levels of mental health knowledge. The mean score of MICA decreased from 47.92 ± 8.63 to 43.53 ± 9.61 after the training (*t* = 6.64, *P* < 0.001). As for the RIBS, the mean scores increased from (14.12 ± 3.90) to (15.38 ± 3.41) at post-test (*t* = -5.44, *P* < 0.001), indicating a significant improvement.

**Conclusions:**

The results from this study show that the training course is an effective way to improve community mental health staff’s attitudes toward people with mental illness in the short term, as well as to lessen the social distance between staff and people with mental illness.

## Background

Most mental disorders are chronic diseases with high prevalence, severity, relapse, disability and have high economic costs. It is reported that mental and substance use disorders accounted for 7.4% of all disability-adjusted life years (DALYs) worldwide in 2010 [1]. Notably, however, more than 70% people with mental illness don’t receive treatment from health staff [2, 3] even though there already are some effective treatments for mental disorders. Almost 76% to 85% people with severe mental disorders in low and middle income countries (LMIC) are untreated for their mental health conditions, and even for high income countries, the corresponding figures are also between 35% and 50% [4]. The scarcity of psychiatrists may be one contributory factor to the prevalent unmet needs in the field of mental health care. WHO reported that the median rate of psychiatrists (per 100,000 populations) ranges from 0.05 for low income countries (LIC) to 8.59 for high income countries [5]. In China, with a total number of 173 million people affected by mental disorders, there are only 1.54 psychiatrists per 100,000 population [6].

To close the enormous treatment gap between the prevalence of mental illness and the available human resources, mental health care has undergone some major changes during the past 50 years. The development of community-based care may be the most important of such changes [7]. Thus, it is the general health care staff, especially primary care staff, who need to take responsibility for managing people with mental disorders in community. There is an international consensus that the delivery of the mental health service at the primary level can reduce the stigma of attending to a psychiatric hospital and a clinic, and it can help early diagnosis, providing more continuously and accessible services and better therapeutic alliances, effective supervision and management [8].

However, as most general health staff are not familiar with identifying and treating people with mental illness, the challenge of task-shifting is the training of the general health staff in community, equipping them with the competence of detecting mental disorders. In developing countries with acute shortages of mental health staff, the training of the general health staff in community is the most pressing priority to meet the unmet need in mental health [9, 10]. Worldwide, Kenya, Iraq, and many other LIC have already carried out related training targeted on non-professional health staff and made several achievements [11–15].

Since 2004, more and more people with mental disorders who are in a stable state are encouraged to receiving service and being managed at community level so that more patients in an acute stage could receive treatment from specialist psychiatrists. China also has initiated the "686" project to improve the community mental heath service [6]. As one of the five national central cities, Guangzhou has developed a range of training courses and now is developing the "Guangzhou model" in the field of community mental health. As in other countries, among those existing community mental health staff in Guangzhou, most of them are general doctors or nurses and they are not familiar with psychiatry. Besides, only a few of them can work exclusively in the mental health department because of the shortage of primary care doctors. The majority of them are in charge of some other chronic disease clinics such as diabetes and hypertension. In our experience, many of these staff do not like to engage in mental health care, and indeed some reject when asked to do so, which makes it difficult to provide continuous services for people with mental disorders. The stigma against working in a mental health environment appears to be a major barrier in these endeavors [16].

Stigma has a negative influence on the severity of symptoms, compliance with treatment, social function, as well as clinical outcomes among people with mental illness [17–22]. It is associated by staff as a mark of shame and disgrace. It is apparent that mental health staff are often treated not as ‘real’ health staff [23], which may increase their stigma as well as reduce their confidence in work. In the long run, stigma can hinder them from proving better mental health service. Importantly, it has been shown that health staff could be a double-edged sword when providing care [23–28]. Their attitudes toward mental health can have substantially detrimental influence on people with mental disorder and their families [24]. Moreover, many anti-stigma campaigns are initiated by mental health staff or health staff [23], and it has become apparent that such interventions need to target on general health staff and mental health staff, as well as the wider population.

In China, people diagnosed with one of the six severe mental disorders, including schizophrenia, bipolar disorder, schizoaffective psychosis, paranoid mental disorders, mental disorders due to epilepsy, or mental retardation, are required to be reported to the relevant health departments according to the National Health and Family Planning Commission’s requirement so as to facilitate their treatment and care. Among these six disorders, schizophrenia and bipolar disorder are the most common. However, the detection rates are low for both in communities probably because most of the health staff in communities are not very familiar with them.

The aims of this study are therefore to improve the community mental health staff’s level of knowledge of mental disorders, especially schizophrenia and bipolar disorder. In addition, we hope to reduce the stigma among the community mental health staff through the training intervention.

## Methods

### Study design

This study was designed to assess a one-day mental health training intervention which was aimed to improve the mental health knowledge (primarily schizophrenia and bipolar disorder) and reduce the stigma among community mental health staff. The participants were asked to complete a questionnaire before and after the training. The study was conducted on March, 2014. Ethics approval was obtained from Research Ethics Committee of Guangzhou Brain Hospital (Number 66, 2013).

### Participants and trainers

The 12 administrative regions in Guangzhou were divided into two groups according to their geographical location, namely a group of inner city and a group of outer city. Then, we chose four districts from each group randomly. Hence, there were 8 regions involved in the study. Then we informed the community mental health staff who worked in the selected districts to participate in the voluntary training. All of them were informed that they could leave the training at any time. A total of 109 participants enrolled in the training. In addition, three trainers from Guangzhou Brain Hospital and one from the first affiliated hospital of Guangzhou Medical University were invited to give classes during the training. All of the trainers have practiced psychiatry for more than ten years.

### The training

The training was comprised of two parts. One included an introduction to mental disorders, primarily schizophrenia and bipolar disorder. The trainers introduced mental disorders in three major areas: symptom, diagnosis and treatment, and other common mental disorders were also introduced briefly. The second part of the training was about stigma related to mental health. The content of this part included:1) What is stigma? 2) The influences of stigma; and 3) How to reduce stigma. At this point, the trainer was also asked to share his own experience related to stigma in the class to help the participants to build confidence in their role.

### Measures

To evaluate the levels of knowledge in participants, we chose two vignettes from the book published by People’s Medical Publishing House (sixth edition). One is schizophrenia [29] and the other is bipolar disorder [29]. Each vignette was followed by three part choice questions: 1) to recognize the symptom in the vignette (multiple choice questions); 2) the appropriate diagnosis in your opinion, and 3) the appropriate treatment.

The Reported and Intended Behavior Scale (RIBS) was used to estimate reported and intended behavioral discrimination related to mental health among the participants [30]. The RIBS is an 8-item scale and items 1-4 only calculate the prevalence of behavior while items 5-8 are used to assess the willingness to engage in the stated behavior. The scale rated on a 5-point scale with a range of 1 = totally disagree to 5 = totally agree. "Don’t know" is anchored at neutral (i.e. 3). The total score is calculated by adding the response values for items 5-8 and can range from 4 to 20. A higher score indicates greater willingness to contact people with mental illness. The Chinese version of RIBS has been reported to have strong internal consistency (Cronbach alpha = 0.82) and test-retest reliability (*r* = 0.68) [31].

The Mental Illness: Clinicians’ Attitudes Scale (MICA) was also included to assess the participants’ attitudes towards mental illness and psychiatry [32]. The scale contains 16 items. Each item is rated on a 6-point scale and responses options are 1 = strongly agree, 2 = agree, 3 = somewhat agree, 4 = somewhat disagree, 5 = disagree, 6 = strongly disagree. Scores can range from 16 to 96 and a lower score indicates low level of stigma. Items 1, 2, 4, 5, 6, 7, 8, 13, 14 and 15 require reverse scoring. The Chinese version of MICA has been reported to have strong internal consistency (Cronbach alpha = 0.72) and test-retest reliability (r = 0.76) [33]. All of the participants were asked to complete the same questionnaire before and immediately after the training.

### Statistical analysis

Data analysis was performed with SPSS version 13. Statistical methods included a general description of quantitative data, paired *t*-testing for continuous variables, and the McNemar test was used to compare dichotomous variables when two time points had to be compared (pre and post test). Participants who didn’t complete both pre-test and post-test were excluded from the analysis. The level of significance was set at 0.05.

## Results

### Participants’ characteristics

Of the 109 participants who attended the study, 99 completed both the pre and post test assessments.Of the 10 others, some participants did not finish the whole training while some others chose not to complete the post-test. The socio-demographic characteristics of the participants were shown in Table 1. The mean age of the participants was 34.8 years with a range of 24 to 59 years. The proportion of male and female were more or less same while male accounted for 51.5% and female for 48.5%. We found that 74.7% of the participants were clinicians, and 18.2% were public health workers.

**Table 1:** **The socio-demographic properties of the participants (n = 99)**

Variable		n	%
Age mean (S.D.) =34.80 (8.38). Range =24-59			
Gender	Male	51	51.5
	Female	48	48.5
Levels of education	Vocational school	7	7.1
	Undergraduate/junior college	88	88.9
	Postgraduate	4	4.0
Marital status	Single	32	32.3
	Married	66	66.7
	Divorced	1	1.0
Job title	Clinician	74	74.7
	Public health worker	18	18.2
	Nurse	5	5.1
	Pharmacist	2	2.0

### Response to mental illness questions in the vignettes

Participants were asked to read the two vignettes and responded to the questions followed. Table 2 showed the change in the recognition of symptom in the vignettes. In the vignette of schizophrenia, prior to the training, 4% participants picked out all the psychotic symptoms presented in the vignette; this number increased to 7.1% after the training (x^2^ = 0.57, *P* = 0.453). With respect to the bipolar disorder vignette, the proportion of people who correctly recognized the psychotic symptoms climbed from 11.1% at the baseline to 22.2% after the training (x^2^ = 4, *P* = 0.043, Cramer Φ = 0.2).

**Table 2:** **Change in the recognition of symptom in the vignettes (n = 99)**

	Pre-course (%)	Post-course (%)	P*
Schizophrenia	4(4.0)	7(7.1)	0.453
Bipolar disorder	11(11.1)	22(22.2)	0.043

*McNemar Test.

Table 3 showed the Change in the recognition of diagnosis in the vignettes. Before the training, 62.6% participants were able to correctly recognize the disorder in the schizophrenia vignette and this number increased to 71.7% after receiving the training intervention (x^2^ = 2.56,*P* = 0.108). Regarding the bipolar disorder vignette, there was a small increase from 73.8% to 76.8% during the two tests (x^2^ = 0.24, *P* = 0.629).

**Table 3:** **Change in the recognition of diagnosis in the vignettes (n = 99)**

	Pre-course (%)	Post-course (%)	P*
Schizophrenia	62(62.6)	71(71.7)	0.108
Bipolar disorder	73(73.8)	76(76.8)	0.629

*McNemar Test.

Concerning treatment, in schizophrenia vignette, 25.3% of the participants chose the appropriate treatment plan before the intervention and the number significantly improved to 62.6% at the end of training(x^2^ = 26.84, *P* < 0.001, Cramer Φ = 0.52). However, with respect to bipolar disorder vignette, there was a modest reduction in the number of participants who could correctly select the most appropriate treatment plan (49.5% vs. 47.5%, *P* = 0.864), see Table 4.

**Table 4:** **Change in the recognition of appropriate treatment in the vignettes (n = 99)**

	Pre-course (%)	Post-course (%)	P*
Schizophrenia	25(25.3)	62(62.6)	<0.001
Bipolar disorder	49(49.5)	47(47.5)	0.864

*McNemar Test.

### Changes in the scores of MICA and RIBS before and after training

The mean score of MICA significantly decreased from 47.92 before the intervention to 43.53 after the training (*t* = 6.64, *df* = 98, *P* < 0.001, Cohen *d* = 0.48). Further, the mean score of RIBS significantly increased from 14.12 to 15.38 between the before and after training (*t* = -5.44, *df* = 98, *P* < 0.001, Cohen *d* = 0.34). More details were shown in Table 5.

**Table 5:** **The mean scores of the MICA and RIBS before and after training in participants (n = 99, Mean ± S.D.)**

	Pre-test	Post-test	***t***	***P***
MICA	47.92 ± 8.63	43.53 ± 9.61	6.64	<0.001
RIBS	14.12 ± 3.90	15.38 ± 3.41	-5.44	<0.001

## Discussion

This was the first study to evaluate the training course on improving the knowledge and attitude toward mental illness among community mental health staff in Guangzhou, China and several benefits were shown in the evaluation. The training changed participants’ attitudes toward mental illness, lessened the social distance between the community mental health staff and people with mental illness, slightly improved the participants’ knowledge level of schizophrenia and bipolar disorder in the vignettes.

Our study indicated that it was possible to train community mental health staff with a short course in stigma related to mental health and to achieve effective outcomes of improved attitudes and behavior. The intervention, however, did not significantly improve knowledge of schizophrenia or bipolar disorder. The short duration of training may be one reason for this, since most of the similar training continued more than three days [11, 12]. Regarding the schizophrenia vignette, before the training, only 4% of the participants could recognize all the symptoms in the vignette and this ratio increased to 7.1% after the training. 71.7% of the participants could identify schizophrenia after the training and this percent was only 62.6% at the baseline. More encouragingly, the proportion of participants who could pick up the appropriate treatment significantly climbed to 62.6% from 25.3%. While, in vignette of bipolar disorder, 11.1% of the participants could recognize all the symptoms in the vignette before the training and this ratio significantly increased to 22.2% after the training. At the baseline, 73.8% of the participants could identify bipolar disorder and this percent was increased to 76.8% after the training. Surprisingly, the percent of participants who could pick up the appropriate treatment dropped to 47.5% from 49.5%. Educational intervention have been proved to have somewhat positive impacts on participants’ opinions on mental illness as well as knowledge related to mental illness [11, 12, 34, 35]. In terms of future study aiming to achieve substantially outcomes of improved knowledge in mental health,the course may need to be longer and long term outcome should also be assessed.

It was rewarding to see the mean score of MICA decreased from 47.92 to 43.53 while the mean score of RIBS increased from 14.12 to 15.38 after the training. And both of them reached a statistical significance. It suggested that through the training, participants had a more positive attitude toward mental disorder and were more willing to contact people with mental disorder after the training. Research had reported the importance of mental health staff in a combat against stigma. The mental health staff’s attitude toward mental disorder had substantial influence on the confidence in treatment, treatment compliance as well as the recovery among patients [23, 24]. Additionally, as a result of integrating mental health into primary care, more and more people with mental illness were receiving treatment at the community level, and community mental health staff were often the first point of contact for people with mental illness. Their stigmatizing attitudes toward mental illness may prevent people with mental illness from receiving the treatment they need [27, 36, 37]. Hence, it was of significance to improve the community mental health staff’s attitude toward mental illness.

### Limitation

This study does have some limitations. Firstly, we did not follow up the participants given that there will be some confounding factors. For example, some of the participants may participate in other related training, which will make it difficult to confidently explain the changes. Secondly, although contacting people with mental disorders was proved to be an effective strategy for reducing stigmatizing attitude about mental disorder [38], it must be acknowledged that in a traditional country like China, mianzi is significantly meaningful to most Chinese people. So people with mental disorders usually remain hidden from the public [39, 40]. Considering these, there are still a wide variety barriers to overcome for people affected by mental disorder or their families to stand out and give a live lecture. Given all the above, we invited a senior psychiatrist to do the lecture instead and made some achievements of combating stigma as well. Thirdly, because most community mental health staff are very busy, we only carried out a one-day training. But it is necessary to equip the community mental health staff with more knowledge about mental disorders through some long-term training. Fourthly, the scales used in the study are all self-reported, so we couldn’t avoid if some participants gave certain responses to please the researchers. Lastly, our study is lack of a control group. As this was an initial and uncontrolled study, future similar studies should be designed with appropriate control or comparison groups.

## Conclusion

The one-day training course changed participant’s attitude toward mental disorder and lessened their distance to people with mental disorder. However, due to the limited time, it was not very effective in terms of improving knowledge of schizophrenia and bipolar disorder. Both the knowledge and attitude are the cornerstone of delivering better mental health services. In order to respond to the call to integrate mental health service into primary care, more and more attention and resources should be invested into community health service. We conclude that, in the future, in the fight to close the treatment gap in mental health and in the combat against stigma related to mental disorder, community mental health staff are going to play a substantial role. Hence, more long-time training sessions, possible with longer-term follow up or booster sessions, may be needed to be developed to improve knowledge and attitudes among community mental health staff.
